# Characterization of the Complete Mitochondrial Genome and Phylogenetic Analyses of *Eurytrema coelomaticum* (Trematoda: Dicrocoeliidae)

**DOI:** 10.3390/genes14122199

**Published:** 2023-12-11

**Authors:** Fuqiang Huang, Xin Li, Bijin Ye, Yule Zhou, Zhisheng Dang, Wenqiang Tang, Long Wang, Haoji Zhang, Wenting Chui, Jun Kui

**Affiliations:** 1 School of Life Science and Engineering, Foshan University, Foshan 528225, China; lixin990730@163.com (X.L.);; 2 National Institute of Parasitic Diseases, Chinese Center for Diseases Control and Prevention (Chinese Center for Tropical Diseases Research), Key Laboratory of Parasite and Vector Biology, National Health Commission of the People’s Republic of China (NHC), World Health Organization (WHO) Collaborating Center for Tropical Diseases, National Center for International Research on Tropical Diseases, Shanghai 200025, China; 3 State Key Laboratory of Hulless Barley and Yak Germplasm Resources and Genetic Improvement, Lhasa 850002, China; 4 Tibet Academy of Agriculture and Animal Husbandry Sciences, Lhasa 850009, China; 5 Animal Disease Prevention and Control Center of Qinghai Province, Xining 810003, China; 6 Huangzhong District Animal Husbandry and Veterinary Station, Xining 811600, China

**Keywords:** *Eurytrema coelomaticum*, mitochondrial DNA, phylogenetic analysis, genome

## Abstract

*Eurytrema coelomaticum*, a pancreatic fluke, is recognized as a causative agent of substantial economic losses in ruminants. This infection, commonly referred to as eurytrematosis, is a significant concern due to its detrimental impact on livestock production. However, there is a paucity of knowledge regarding the mitochondrial genome of *E. coelomaticum*. In this study, we performed the initial sequencing of the complete mitochondrial genome of *E. coelomaticum*. Our findings unveiled that the mitochondrial genome of *E. coelomaticum* spans a length of 15,831 bp and consists of 12 protein-coding genes, 22 tRNA genes, two rRNA genes, and two noncoding regions. The A+T content constituted 62.49% of the genome. Moreover, all 12 protein-coding genes of *E. coelomaticum* exhibit the same arrangement as those of *E. pancreaticum* and other published species belonging to the family Dicrocoeliidae. The presence of a short string of additional amino acids (approximately 20~23 aa) at the N-terminal of the cox1 protein in both *E. coelomaticum* and *E. pancreaticum* mitochondrial genomes has contributed to the elongation of the *cox1* gene in genus *Eurytrema*, surpassing that of all previously sequenced Dicrocoeliidae. The phylogenetic analysis displayed a close relationship between *E. coelomaticum* and *E. pancreaticum*, along with a genus-level association between *Eurytrema* and *Lyperosomum*. These findings underscore the importance of mitochondrial genomic data for comparative studies of Dicrocoeliidae and even Digenea, offering valuable DNA markers for future investigations in the systematic, epidemiological, and population genetic studies of this parasite and other digenean trematodes.

## 1. Introduction

Eurytrematosis, also known as pancreaticosis, is caused by parasitic trematodes belonging to the genus *Eurytrema*. These trematodes infect the pancreatic ducts of cattle, sheep, and other ruminants. In addition to infecting these livestock, certain species of these parasites can also infect humans [1,2]. Worms that stimulate the pancreatic duct can cause chronic proliferative inflammation and progressive necrosis of the mucosal epithelium. This, in turn, leads to the thickening of the tube wall, narrowing of the lumen, and possible occlusion [3,4]. Currently, over ten species of genus *Eurytrema* have been reported in the world, including *E. pancreaticum*, *E. coelomaticum*, *E. cladorchis*, *E. dajii*, *E. ovis*, *E. tonkinense*, *E. parvum*, and *E. fukienensis* [5]. However, some species within the genus *Eurytrema* have limited written descriptions, lacking anatomical structure maps, explanations of their life history, as well as other relevant information, such as gene sequences. Thus, there is a controversy regarding the validity for most of the type species [6]. *E. pancreaticum*, *E. coelomaticum*, and *E. cladorchis* are generally regarded as valid species and are considered the most predominant species in ruminants in China. These three parasites can coexist in the same region, especially in southern China. This coexistence creates difficulties in accurately identifying the *Eurytrema* species in the same region [7].

The mitochondrial genome possesses a distinctive genetic sequence that is inherited exclusively from the maternal line and exhibits a straightforward structure [5]. Due to its highly conserved coding region, elevated copy number, and limited recombination rate, the mitochondrial genome serves as a frequently utilized molecular marker in the examination of biological system evolution [8]. Its wide array of applications includes animal phylogeny, species identification, and parasite diagnosis, among other fields [9,10]. Up to date (December 2023), the GenBank database currently contains information only on three confirmed species within the *Eurytrema* genus: *E. pancreaticum*, *E. coelomaticum*, and *E. cladorchis*. Among these three species, only *E. pancreaticum* has an available complete mitochondrial genome sequence. As for other *Eurytrema* species, such as *E. coelomaticum*, sequencing has been limited to a few nuclear RNAs, including the 18S RNA gene. To accurately identify *E. coelomaticum* and provide additional molecular resources for further studies on *Eurytrema* taxonomy, population genetics, and systematics, we employed the Illumina NovaSeq sequencing platform and utilized the whole-genome shotgun strategy to create a library of the mitochondrial genome of *E. coelomaticum*.

## 2. Materials and Methods

### 2.1. Identification of Eurytrema coelomaticum

Samples were collected from the pancreatic organs of goats admitted to the Veterinary Hospital of Foshan University. The samples were stored in 70% alcohol at 4 °C. An adult worm was randomly selected, and its size was measured using a Vernier caliper. It was then stained with carmine. Its morphological characteristics were observed under a microscope, leading to its identification as *E. coelomaticum* (Figure S1) based on the recently published morphological structure [11]. Additionally, it was identified as *E. coelomaticum* through the utilization of the 18S RNA and *cox1* gene, as described in our previous study [12].

### 2.2. DNA Extraction and Sequencing

Genomic DNA extraction was performed using a genomic extraction kit provided by Nanjing Novizan Company on approximately 30 mg of parasite tissue that was cut into pieces. The concentration and purity of DNA were measured using a Thermo Scientific NanoDrop 2000 (Thermo Fisher Scientific Inc, the US), while DNA integrity was assessed using agarose gel electrophoresis and an Agilent 2100 Bioanalyzer. DNA samples that passed quality checks were subjected to shearing and fragmentation using a Covairs machine. Additionally, the ends of the DNA fragments were repaired using exonuclease and polymerase functions. The 3′ end of the splice contained a single T base, while a single A base was introduced into the 3′ end, allowing for complementary pairing of A and T to connect DNA fragments and splices. The splice containing the tag was incubated with ligase to connect it to the DNA fragment. The DNA fragments with connectors were then selectively enriched, and the DNA library was subsequently amplified. The library size was determined using an Agilent 2100 Bioanalyzer, while the total concentration of the library was measured through fluorescence quantitation. A single-chain library served as a template for bridging the polymerase chain reaction (PCR) amplification, synthesis, and sequencing. To validate the next-generation sequencing (NGS) result, PCR was conducted on the extracted DNA to amplify the cox1 gene, followed by Sanger sequencing. All polymerase chain reactions (PCRs) were conducted using the Taq PCR Master Mix Kit (Sangon Biotech, Shanghai, China) and a thermal cycler (Biometra TAdvanced 96 SG, Jena, Germany). Two sets of primers were used: 1) COX1A-F (5′-AGGTTAGGAGAGACTGTCTG-3′) and COX1A-R (5′-ACAAGCTGGAGCCAACAATC-3′), and 2) COX1B-F (5′-GTGTCTCCAGGTTTGATTCC-3′) and COX1B-R (5′-CGAATATCACACCCTACCAAC-3′). The PCR protocol consisted of an initial denaturation step at 94 °C for 3 min, followed by 36 cycles at 94 °C for 30 s, 52 °C for 40 s, and 72 °C for 45 s. A final extension was performed at 72 °C for 10 min.

### 2.3. Genome Sequence Assembly and Analysis

A5—Miseq v20150522 [13] and SPAdesv3.9.0 [14] were utilized for the assembly of the NGS data, starting from the initial stage, and for the construction of the contig and scaffold sequences. Sequences were extracted by considering the sequencing depth of the stitched sequences. Subsequently, sequences with high sequencing depths were subjected to BLASTN analysis using the nt library on NCBI (BLAST v2.2.31+), enabling the identification of mitochondrial sequences from each stitching result. By referring to the reference sequence and utilizing mummers v3.1 software, collinearity analysis was conducted to ascertain the positional relationships between contigs and facilitate contig gap filling [15]. The final mitochondrial sequence was obtained through the utilization of pilon v1.18 [16] to correct the obtained results.

### 2.4. Genome Annotation

The complete mitochondrial genome sequence was uploaded to MITOS for initial annotation and prediction of the secondary structure of RNA genes [17]. Additionally, the annotation results were manually corrected using SnapGene (V6.0.2) software and validated through homology alignments with other trematode species.

### 2.5. Phylogenetic Analysis

Mitochondrial genomes play a crucial role in the systematic classification of species. The combination of multiple genes can enhance the stability and reliability of phylogenetic reconstruction. To compare and analyze the mitochondrial genome dataset of 59 trematode species (Table S1), we utilized the Phylosuite [18]. To ensure the accuracy of names and annotations, we initially normalized the sequences using both Phylosuite and manual approaches. We aligned the protein-coding genes using the MAFFT program [19] in normal alignment mode. After removing ambiguously aligned regions using Gblocks 0.91b [20], we concatenated the alignment results of all protein-coding genes into a joint dataset in PhyloSuite. Subsequently, the resulting files were analyzed using ModelFinder [21] to identify the most suitable evolutionary models for each gene, facilitating the maximum likelihood (ML) phylogenetic analyses. The maximum likelihood phylogeny was inferred using IQ-TREE [22] with the Partition model [23] of nucleotide substitution, and ultrafast bootstraps [24] with 100,000 replicates were performed. Additionally, the phylograms were visualized and annotated using iTOL v5 [25].

## 3. Results

### 3.1. Mitogenome Structure and Nucleotide Composition

**Figure 1:**
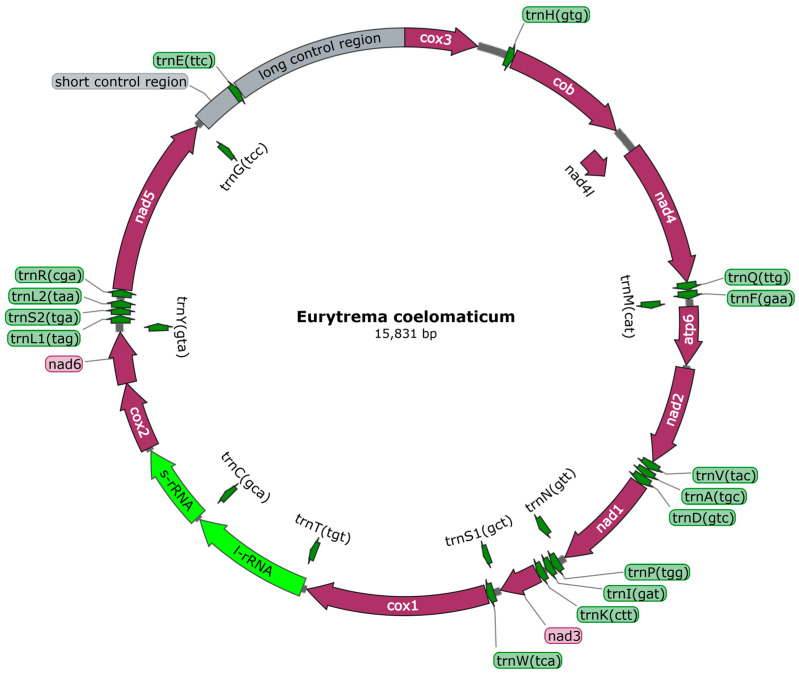
*Eurytrema coelomaticum* mitochondrial genome arrangement. All genes have a standard nomenclature. Twenty-two tRNA genes are designated by a letter code corresponding to amino acids and digitally distinguish between two specified leucine and serine tRNAs.

The raw read sequence obtained through high-quality sequencing was 22,801,366 bp, and the clean read sequence obtained after filtration was 18,207,918 bp. Through gap sequencing, gene splicing, and assembly, we obtained the complete mitochondrial genome sequence of *E. coelomaticum*, which was 15,831 bp in length (GenBank accession: ON297668). The proportions of A, T, C, and G were 18.8%, 43.7%, 11.9%, and 25.6%, respectively, and the A+T content in *E. coelomaticum* was 62.5% (Table 1). The genome annotation results revealed that the genome consisted of 36 genes, including 12 protein-coding genes (PCGs), two rRNA genes, and 22 tRNA genes. Additionally, there were two noncoding regions (Figure 1). The mitochondrial genome of *E. coelomaticum* contained 24 gene spacers in the genome sequence, ranging in length from 1 to 371 bp. Additionally, there were nine overlapping genes with lengths ranging from 1 to 63 bp (Table 2).

### 3.2. Protein-Coding Genes and Codon Usage

**Figure 2:**
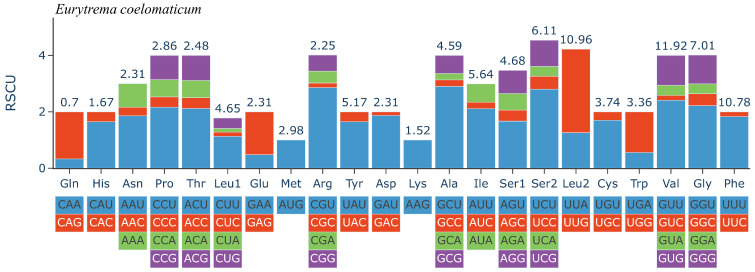
Relative synonymous codon usage (RSCU) of the complete mitochondrial genome of *Eurytrema coelomaticum*. Codon families are labelled on the x-axis. Values on the top of the bars refer to amino acid usage.

**Figure 3:**
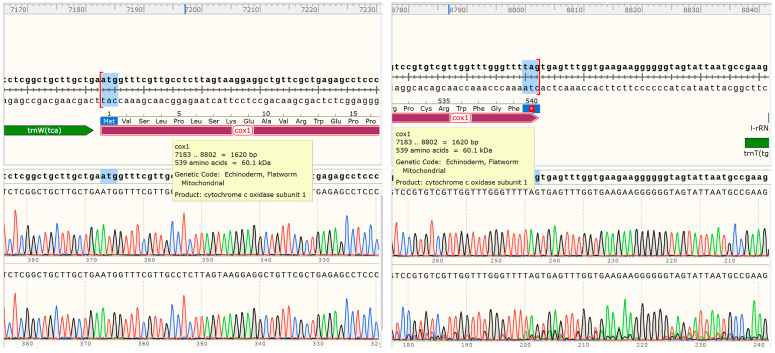
The *cox1* gene location and length in *E. coelomaticum* mitochondrial genome.

The 12 PCGs have a combined length of 10,302 bp, all oriented in the same translation direction and located on the sense sequence (Table 1). Among the protein-coding genes, the *cox1* gene is the longest, spanning 1579 bp (7183–8802), followed by the *nad5* gene, which spans 1617 bp (12,159–13,775). The *nad4L* gene is the shortest, covering 264 bp (2107–2370). Nucleotide codon usage and codon family proportion are presented in Table 1 and the top of the bar of Figure 2, and it is shown that the usage of T is very high and leucine (10.96% + 4.65% = 15.61%), Valine (11.92%), and phenylalanine (10.78%) were the most frequent amino acids in the PCGs of *E. coelomaticum*. The initiation codons of ten PCGs (*nad5, nad6, cox1, nad3, nad1, nad2, atp6, nad4, nad4,* and *cob*) are ATG, while the initiation codons of two PCGs (*cox2* and *cox3*) are GTG. The stop codons of eight PCGs (*atp6, cox2, cob, nad1, nad2, nad3, nad4l,* and *nad6*) are TAA, the stop codons of four PCGs (*cox1, cox3, nad4,* and *nad5*) are TAG (Table 2). To validate the NGS results and confirm the structure of the *cox1* gene, two primers were designed for sequencing the start and end regions of the *cox1* gene. It is shown that the *cox1* gene starts at 7183 bp and ends at 8802 bp, encoding 539 aa (Figure 3). The length of the *cox1* gene in the *E. coelomaticum* mitochondrial genome is the third longest among all currently sequenced family Dicrocoeliidae due to the presence of an additional amino acid tract at the N-terminal. A similar phenomenon is found in *E. pancreaticum*, which belongs to the same genus as *E. coelomaticum* (Figure S3).

### 3.3. tRNA and rRNA Genes

A total of 22 tRNAs, ranging in size from 60 to 80, were annotated in *E. coelomaticum*, with a total gene length of 1476 bp (Table 1). The tRNA structures were predicted by MITOS, revealing that the structures of tRNA-S1 and tRNA-S2 were abnormal, presenting unorthodox structures in which the D-arms were unpaired (Figure S2). The remaining tRNAs exhibited the classical clover structure. The two rRNA genes are situated between the *cox2* gene and the *trnT* gene, with a *trnC* gene separating them (Figure 1). The length of the *rrnL* gene is 1098 bp, while the *rrnS* gene is 722 bp in length. The A+T content of the rRNA gene sequence is 60.8% (Table 1). Two noncoding regions (NCRs) with significant size differences were annotated in the mitochondrial genome of *E. coelomaticum*. The long noncoding region (LNCR) is 1564 bp in length and is situated between the *trnE* gene and the *cox3* gene. The short noncoding region (SNCR) is 371 bp in length and is located between the *trnG* gene and the *trnE* gene. (Figure 1; Table 2). The A+T contents of NCRs are 66% and 69.7%, respectively (Table 1).

### 3.4. Phylogenetic Analysis

**Figure 4:**
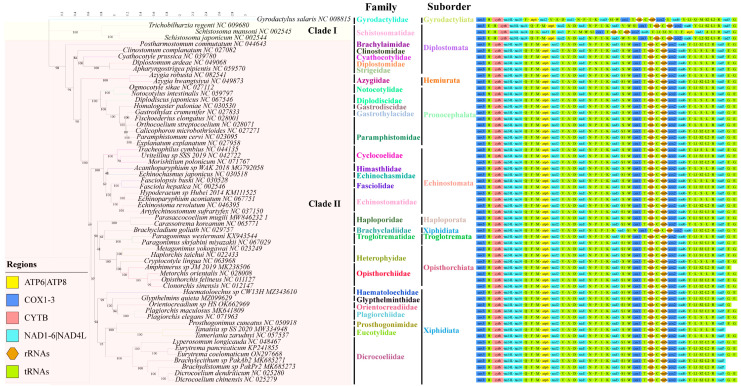
Phylogenetic relationships between *Eurytrema coelomaticum* and other trematodes based on mitochondrial sequences. Using the mitochondrial genome sequences of *Gyrodactylus salaris* (NC008815) as outgroup, the phylogenetic tree was constructed based on 12 protein-coding genes by the maximum likelihood (ML) method. The mitochondrial genome sequences of *Brachylecithum* sp. PakAb2, *Brachydistomum* sp. PakPr2, *Tanaisia* sp. SS-2020, *Orientocreadium* sp. HS, and *Amphimerus* sp. JM-2019 are incomplete.

The phylogenetic tree is constructed using the 12 PCGs from the concatenated regions of the mitochondrial genomes of 59 trematodes (Table S1), with *Gyrodactylus salaris* (NC008815) serving as an outgroup. The maximum likelihood algorithm was used on the basis of the 9803 nucleic acid alignment length, available after Gblocks [20] processing. The phylogenetic tree analysis revealed that the 58 digeneans used in this study, including the suborders Diplostomata, Echinostomata, Haploporata, Hemiurata, Opisthorchiata, Pronocephalata, Troglotremata, and Xiphidiata, could be classified into two major clades. Clade I consists of three representative species of the family Schistosomatidae, while Clade II comprises 55 representatives from 28 families. Clade II includes *E. coelomaticum* and *E. pancreaticum* of the family Dicrocoeliidae, which are closely clustered and form a small clade (Figure 4). According to the phylogenetic tree, among the Digenea of Trematoda, the family Dicrocoeliidae is closely related to the families Prosthogonimidae, Eucotylidae, and clusters further with the families Plagiorchiidae, Orientocreadiidae, Haematoloechidae, and Glypthelminthidae within the suborder Xiphidiata. Additionally, based on the complete mitochondrial genome sequences of the used Digeneans, the genus *Eurytrema* is closely related to the genus *Lyperosomum* within the family Dicrocoeliidae (Figure 4). The entire family Dicrocoeliidae exhibits close clustering, despite significant evolutionary divergence.

## 4. Discussion

The species of the genus *Eurytrema* are common flukes in ruminants. However, the mitochondrial genome of some species within the genus *Eurytrema* are not well understood. Many gaps remain in the classification and evolutionary branching of *Eurytrema*. In this study, we sequenced and analyzed the complete mitochondrial genome sequence of *E. coelomaticum*.

The mitochondrial genome of *E. coelomaticum* has a total length of 15,831 bp, which is slightly longer than that of *E. pancreaticum* (15,031 bp) and most other flukes (e.g., 14,014 bp for *Paramphistomum cervi* (Accession: NC_023095.1) and 15,258 bp for *Metagonimus yokogawai* (Accession: NC_023249.1)). However, it falls within the range of mitochondrial genome lengths observed in sequenced trematodes. Notably, it is shorter than *Schistosoma spindale* (16,901 bp, Accession: NC_008067.1) and *Tamerlania zarudnyi* (16,188 bp, Accession: NC_057537.1). The complete mitochondrial genome of *E. coelomaticum* consists of 12 PCGs, two rRNAs, 22 tRNAs, and two noncoding regions. The initiation codons of PCGs in *E. coelomaticum* are similar to those found in *E. pancreaticum* and other Digenea trematodes, with the allocation of stop codons differing, using TAGs and TAAs [26,27]. Additionally, *E. pancreaticum* exhibits some PCGs with abbreviated T stop codons, whereas the sequenced mitochondrial genome of *E. coelomaticum* does not demonstrate this phenomenon. While it is common among metazoan mitochondrial protein genes, such abbreviated T stop codons have only been identified in cestodes among flatworms [26]. As for the *cox1* gene, the maximum percentage variation in its protein size is 3.5% (512 aa–530 aa) in the mitochondrial DNA (mtDNA) of vertebrates and higher invertebrates such as insects and echinoderms [9]. The sequenced *cox1* genes of trematodes in the RefSeq database show a range of sizes, from 510 aa (Fasciola hepatica, Accession: NP_066225) to 684 aa (Tamerlania zarudnyi, Accession: YP_010166578.1). Furthermore, within the genus *Schistosoma*, there is significant variation in the size of the *cox1* gene, ranging from 513 aa to 609 aa [26,28,29]. In addition, the previous study shows that the structural abnormalities of tRNA-S1 and tRNA-S2 are normal in flatworms [30]. The long noncoding region (NCR) of *E. pancreaticum* is 989 bp in length [31], while that of *E. coelomaticum* measures 1564 bp. Trematodes of the same genus exhibit significant variations in NCR length, possibly due to the presence of a substantial number of repeats in the NCR of the mitochondria [32,33]. To address the issue of inaccurate NCR annotation, additional advancements in sequencing technology are required. The gene order of protein-coding and ribosomal RNA genes in the mitochondrial genome of *E. coelomaticum* conforms to the characteristic pattern observed in digeneans [30]. Furthermore, it was demonstrated that the positions of the last two genes, namely tRNA-Glu and tRNA-Gly, were interchanged across different species within Digenea, except for Schistosomatidae and the species of *Brachycladium goliath* (Figure 4).

In the past, studies on *Eurytrema* and even Dicrocoeliidae have mainly focused on morphology, life history, and prevalence, but little on the genetic evolution of genes. For decades, there has been considerable controversy over the taxonomy of Digenea. Previous studies on the phylogenetic analysis of Digenea and *E. coelomaticum* based on 28S rDNA and 18S rDNA sequences yielded different results [5,34,35]. Studies have shown that the mitochondrial genome sequence may provide useful genetic markers for examining the classification status of trematodes, especially when protein-coding gene sequences are used as comparative analysis markers [36,37]. In this study, phylogenetic analysis of mitochondrial genome sequences strongly suggests that the phylogeny of Digenea can be classified into two major clades. One clade consists of three species of Schistosomatidae, while the other consists of 55 members from 28 different families. Consistent with previous research [38], the evolutionary tree constructed in this study suggests that the suborders Xiphidiata and Diplostomata are paraphyletic, while other suborders are monophyletic and rooted in single branches. Additionally, in the Diplostomata branch, the families Clinostomidae, Cyathocotylidae, Dipostomidae, and Strigeidae cluster together, instead of being grouped with Schistosomatidae or Brachylaimidae (Figure 4). These results indicate that the Brachylaimidae, Clinostomidae, Cyathocotylidae, Dipostomidae, and Strigeidae taxonomies require further study. Within the suborder Xiphidiata, *E. coelomaticum* and *E. pancreaticum* are clustered together and are sister to *Lyperosomum longicauda* that form a small clade. This clade is more closely related to Dicrocoelium chinensis, *D. dendriticum*, *Brachylecithum* sp., and *Brachydistomum* sp. in the family Dicrocoeliidae than it is to species in Plagiorchiidae and Orientocreadiidae. Although Brachycladiidae is currently classified within the suborder Xiphidiata, an evolutionary tree constructed based on the species of *Brachycladium goliath* shows a closer relationship to Troglotrematidea, which is consistent with previous research, and indicated that Brachycladiidae and Troglotrematidae are more closely related to each other than either are to Dicrocoeliidae and Plagiorchiidae [38,39,40].

Our sequencing and annotation of the complete mitochondrial genome of *E. coelomaticum* makes a significant contribution to the Digenea database, specifically regarding the genus *Eurytrema*. Building upon these efforts, we reconstructed the phylogenetic tree of Digenea, validating the evolutionary relationships between *E. coelomaticum* and various Digenea flukes. This enhances our understanding of the overall phylogenetic relationship of Digenea.

## 5. Conclusions

This is the first reported complete mitochondrial genome of *E. coelomaticum.* The mitochondrial genome composition of *E. coelomaticum* is highly similar to that of *E. pancreaticum*, including 36 genes and two noncoding regions. Phylogenetic analysis showed that species of the genus *Eurytrema* and the genus *Lyperosomum* are more closely related to each other than to other trematodes. The mitochondrial data of *E. coelomaticum* can be a valuable resource for studying the evolutionary relationships within the genus *Eurytrema* and even within the family Dicrocoeliidae. Furthermore, it can provide a basis for the detection and systematic analysis of this parasite.

## Figures and Tables

**Table 1:** *Eurytrema coelomaticum* mitochondrial complete genome, protein coding genes, and rRNA gene base composition.

Regions	Strand	Size (bp)	T(U) (%)	C (%)	A (%)	G (%)	A+T (%)	G+C (%)	AT Skewness	GC Skewness
Full genome	+	15,831	43.7	11.9	18.8	25.6	62.5	37.5	−0.399	0.365
PCGs	all	10,302	47	11.7	15.8	25.5	62.8	37.2	−0.497	0.37
PCGs	+	10,302	47	11.7	15.8	25.5	62.8	37.2	−0.497	0.37
tRNAs	all	1476	36.8	13.9	21.1	28.2	57.9	42.1	−0.27	0.34
tRNAs	+	1476	36.8	13.9	21.1	28.2	57.9	42.1	−0.27	0.34
rRNAs	all	1820	38.2	12.4	22.6	26.8	60.8	39.2	−0.256	0.366
rRNAs	+	1820	38.2	12.4	22.6	26.8	60.8	39.2	−0.256	0.366
1st codon position	all	3434	40.3	12.1	19.5	28	59.8	40.1	−0.347	0.398
1st codon position	+	3434	40.3	12.1	19.5	28	59.8	40.1	−0.347	0.398
2nd codon position	all	3434	46.8	16	16.3	21	63.1	37	−0.484	0.135
2nd codon position	+	3434	46.8	16	16.3	21	63.1	37	−0.484	0.135
3rd codon position	all	3434	53.9	7.1	11.6	27.5	65.5	34.6	−0.647	0.59
3rd codon position	+	3434	53.9	7.1	11.6	27.5	65.5	34.6	−0.647	0.59
atp6	+	519	52.8	11.6	11.2	24.5	64	36.1	−0.651	0.358
cox1	+	1620	44.4	13.8	16.6	25.2	61	39	−0.456	0.293
cox2	+	609	43	13.8	17.4	25.8	60.4	39.6	−0.424	0.303
cox3	+	651	46.7	12.3	16.4	24.6	63.1	36.9	−0.479	0.333
cytb	+	1119	46.7	12.2	16.5	24.5	63.2	36.7	−0.477	0.333
nad1	+	933	45.3	10.5	16.6	27.5	61.9	38	−0.464	0.448
nad2	+	879	49.8	9.9	14.2	26.1	64	36	−0.556	0.449
nad3	+	357	50.4	8.4	15.1	26.1	65.5	34.5	−0.538	0.512
nad4	+	1278	48	10.9	13.7	27.4	61.7	38.3	−0.556	0.431
nad4L	+	264	45.8	9.5	18.2	26.5	64	36	−0.432	0.474
nad5	+	1617	46.5	12.4	17.5	23.6	64	36	−0.453	0.309
nad6	+	456	50.7	9.4	13.6	26.3	64.3	35.7	−0.577	0.472
rrnL	+	1098	39.9	11.9	22.3	25.9	62.2	37.8	−0.283	0.369
rrnS	+	722	35.6	13.2	23.1	28.1	58.7	41.3	−0.212	0.362
SNCR	/	371	41.5	11.6	27.2	19.7	69.7	31.3	−0.208	0.259
LNCR	/	1564	35	11,6	31	22.4	66	34	−0.061	0.318

+: sense strand (coding strand).

**Table 2:** *Eurytrema coelomaticum* mitochondrial genome organization.

Feature	Position	Length (bp)	Initiation Codon	Stop Codon	Anticodon	Intergenic Nucleotide
*cox3*	1–651	651	GTG	TAG		266
*trnH*	918–987	70			GTG	1
*cob*	989–2107	1119	ATG	TAA		−1
*nad4l*	2107–2370	264	ATG	TAA		−40
*nad4*	2331–3608	1278	ATG	TAG		3
*trnQ*	3612–3676	65			TTG	11
*trnF*	3688–3757	70			GAA	−3
*trnM*	3755–3822	68			CAT	3
*atp6*	3826–4344	519	ATG	TAA		27
*nad2*	4372–5250	879	ATG	TAA		10
*trnV*	5261–5325	65			TAC	10
*trnA*	5336–5398	63			TGC	5
*trnD*	5404–5477	74			GTC	3
*nad1*	5481–6413	933	ATG	TAA		−29
*trnN*	6385–6464	80			GTT	10
*trnP*	6475–6545	71			TGG	0
*trnI*	6546–6610	65			GAT	18
*trnK*	6629–6694	66			CTT	1
*nad3*	6696–7052	357	ATG	TAA		−12
*trnS1*	7041–7100	60			GCT	13
*trnW*	7114–7181	68			TCA	1
*cox1*	7183–8802	1620	ATG	TAG		35
*trnT*	8838–8904	67			TGT	−63
*rrnL*	8842–9939	1098				−41
*trnC*	9899–9964	66			GCA	1
*rrnS*	9966–10,687	722				29
*cox2*	10,717–11,325	509	GTG	TAA		0
*nad6*	11,326–11,781	456	ATG	TAA		−1
*trnY*	11,781–11,853	73			GTA	7
*trnL1*	11,861–11,926	66			TAG	10
*trnS2*	11,937–11,996	60			TGA	2
*trnL2*	11,999–12,062	64			TAA	29
*trnR*	12,092–12,155	64			CGA	3
*nad5*	12,159–13,775	1617	ATG	TAG		−10
*trnG*	13,766–13,831	66			TCC	371
SNCR	13,832–14,202	371				0
*trnE*	14,203–14,267	65			TTC	0
LNCR	14,268–15,831	1564				0

## Data Availability

GenBank Accession ON297668.

## Supplementary Materials

The following supporting information can be downloaded at: https://www.mdpi.com/article/10.3390/genes14122199/s1, Figure S1: Morphology of the adult of *Eurytrema coelomaticum* from goat in Guangdong; Figure S2: Inferred secondary structures of the trnS1 and trnS2 from *Eurytrema coelomaticum*; Figure S3: Alignment of the *cox1* protein sequences from trematode, nematodes, and *Homo sapiens*; Table S1: Genes sequence used for phylogenetic analysis [41].
