# Supplementary material for: Characterization of the Complete Mitochondrial Genome and Phylogenetic Analyses of Eurytrema coelomaticum (Trematoda: Dicrocoeliidae)
Source: Genes (Basel). 2023 Dec 11;14(12):2199. doi: 10.3390/genes14122199 (PMC10743053; doi:10.3390/genes14122199)
Supplement: Supplementary file 1 [file genes-14-02199-s001.zip › Figure S3.pdf]

```
1 10 20
Homo_sapiens_P00395 MF......................................ADRWLFSTNHKDIGTLYLLF
Caenorhabditis_elegans_P24893 MNLYK..............................KYQGGLAVWLESSNHKDIGTLYFIF
Ascaris_suum_P24881 MSGFY..............................KYQGGLSVWLESSNHKDIGTLYFLF
Lyperosomum_longicauda_QIX04657 MW....................................LGALAWFFSLDHKRISVIYMIL
Brachydistomum_sp__PakPr2_QIX04645 MSIL.................................WLLSFSWFMSLDHKRISMIYFML
Brachylecithum_sp__PakAb2_QIX04633 LYIS..................................LGTLSWFFSLDHKRISVVYLIL
Dicrocoelium_dendriticum_AHG06504 M........................................FGWFMSLDHKRISMIYFML
Dicrocoelium_chinensis_AHG06492 M........................................FGWFMSLDHKRISMIYFML
Eurytrema_coelomaticum_ON297668 MVSLPLSKE...AVRWE.PPVLLSF........SWTLTVNSVLWFFTLDHKRVSMIYSIL
Eurytrema_pancreaticum_AKC58423 MPLSPLLKE...AVLWEEPPVLFSF........SWSLTLNSVLWLFTLDHKRVSMIYLIL
Schistosoma_spindale_YP_626439 MAL..................................NNGYIYWFISLDHKRIGLIYMFL
Schistosoma_haematobium_YP_626526 MNV...................................VGYGYWLISLDHKRISFIYVIL
Schistosoma_mekongi_AAG12187 MSWRNNLYELYLKILYSLIYLMYRFRWWLEDQLKLENNKDLVIWFVSLDHKRIGIIYMIM
Schistosoma_japonicum_AAG13143 MDWRKNLYELYLKILYSLIYLMYRLRWWIEDQFKYENNKDLISWFVSLDHKRVGVVYIIL
Cotylurus_marcogliesei_AYH51378 MN.....................................TICSWLFTLDHKRIGIIYMIM
Prosthogonimus_cuneatus_YP_009938512 MS......................................LFPWLFSLNHKRIGVMYAVI
Diplostomum_ardeae_YP_009867153 MD.....................................SLYSWLFTLDHKRIGIIYSVI
Paragonimus_westermani_NP_059465 M........................................FTWLFTLDHKRIGMIYMVL
Paramphistomum_cervi_YP_008963803 MV....................................NKVVVWACTMDHKRVGFIYLVV
Ogmocotyle_sikae_YP_009139763 MS......................................GLVWLFTMDHKRVGFIYFVL
Fasciola_hepatica_COX1_15504 M.........................................SWLFTLDHKRVGLIYMVI
Fasciola_gigantica_YP_009026843 MN......................................YISWLFTLDHKRVGLIYMLI
Fischoederius_elongatus_YP_009169430 MS.....................................KVVVWFCTVDHKRVGFIYLVI

30 40 50 60 70 80
Homo_sapiens_P00395 GAWAGVLGTALSLLIRAELGQPGNLLGNDHIYNVIVTAHAFVMIFFMVMPIMIGGFGNWL
Caenorhabditis_elegans_P24893 GLWSGMVGTSFSLLIRLELAKPGFFLSNGQLYNSVITAHAILMIFFMVMPTMIGGFGNWL
Ascaris_suum_P24881 GLWSGMVGTSLSLVIRLELAKPGLLLGSGQLYNSVITAHAILMIFFMVMPTMIGGFGNWM
Lyperosomum_longicauda_QIX04657 GVWGGFLGLGLSMLIRMNFLDPFHNVVSPEVYNYVITNHGIAMIFFFLMPVLIGGFGNYL
Brachydistomum_sp__PakPr2_QIX04645 GLWGGLTGLALSMLIRLNFLDPFHNIVSPEVYNFVVTNHGIAMIFFFLMPVLIGGFGNYL
Brachylecithum_sp__PakAb2_QIX04633 GLWGGFMGLSLSMLIRLNFLDPFYNVVSPEVYNYVITNHGIAMIFFFLMPVLIGGFGNYL
Dicrocoelium_dendriticum_AHG06504 GLWGGLTGLALSMLIRLNFLDPFHNIVSPEVYNYVVTNHGIAMIFFFLMPVLIGGFGNYL
Dicrocoelium_chinensis_AHG06492 GLWGGLTGLALSMLIRLNFLDPFHNIVSPEVYNYVVTNHGIAMIFFFLMPVLIGGFGNYL
Eurytrema_coelomaticum_ON297668 GIWGGFLGLALSMLIRINFLDPFYNIVSPEVYNFVITEHGVAMIFFFLMPVLIGGFGNYL
Eurytrema_pancreaticum_AKC58423 GLWGGFLGLALSMLIRVNFLDPFYNIVSPEVYNFVITEHGVAMIFFFLMPVLIGGFGNYL
Schistosoma_spindale_YP_626439 GIWGGFIGLGLSLLIRLNLCDPYYNLVSMDIYNFLITNHGIAMIFFFLMPVLIGGFGNYF
Schistosoma_haematobium_YP_626526 GLWGGFIGLGLSLLIRLNLCDPYYNLVSLDVYNFLITNHGIAMIFFFLMPILIGGFGNYF
Schistosoma_mekongi_AAG12187 GIWGGFIGLGLSMLIRLNFCDPYYNLIPCEIYNYLITNHGIAMIFFFLMPLLIGGFGNYF
Schistosoma_japonicum_AAG13143 GVWGGFIGLGLSLLIRLNFCDPYYNLIPCEVYNYLITNHGIAMIFFFLMPVLIGGFGNYL
Cotylurus_marcogliesei_AYH51378 GLWGGILGLSLSFLIRLNFADPYYNLISPEVYNYVITSHGLAMIFFFLMPVIVGGFGNFL
Prosthogonimus_cuneatus_YP_009938512 GVWGGFLGLGLSMVIRMNFLNPYSNVVSAEVYNYVVTTHGIAMIFFFLMPVLIGGFGNYL
Diplostomum_ardeae_YP_009867153 GLWGGFMGLSLSFLLRLNFTDPYYNLIAPEVYNYVITSHGLAMIFFFLMPVLIGGFGNFL
Paragonimus_westermani_NP_059465 GIWGGFLGLSLSMLIRLNYLDPYYNIVSSEVYNYVLTNHGVAMIFFFLMPVLIGGFGNYL
Paramphistomum_cervi_YP_008963803 GIWSGFLGLALSTLIRLNYMEPYYNVVSPEVYNYVVSIHGIVMLLFFLMPVLIGGFGNYL
Ogmocotyle_sikae_YP_009139763 GVWGGFLGLSLSMLIRLNFVEPYYNVVAPEVYNYVITIHGVTMIFFFLMPVLIGGFGNYL
Fasciola_hepatica_COX1_15504 GLWGGFFGLSLSVLVRLNYLDPYFNLVSPEVYNYVVTGHGIIMIFFFLMPVLIGGFGNYL
Fasciola_gigantica_YP_009026843 GLWGGFFGLSLSILVRLNYLDPYFNLVSPEVYNYVVTGHGVIMIFFFLMPVLIGGFGNYL
Fischoederius_elongatus_YP_009169430 GIWAGFLGLALSTLIRLNFMEPYYNVISPEVYNYVVSIHGIVMLLFFLMPILIGGFGNYL

90 100 110 120 130 140
Homo_sapiens_P00395 VPLMIGAPDMAFPRMNNMSFWLLPPSLLLLLASAMVEAGAGTGWTVYPPLAGNYSHP.GA
Caenorhabditis_elegans_P24893 LPLMLGAPDMSFPRLNNLSFWLLPTSMLLILDACFVDMGCGTSWTVYPPLSTM.GHP.GS
Ascaris_suum_P24881 LPLMLGAPDMSFPRLNNLSFWLLPTAMFLILDACFVDMGCGTSWTVYPPLSTM.GHP.GG
Lyperosomum_longicauda_QIX04657 LPLFLGLKDLSLPRINALSVWLLVPSCVCLCVSMF..SGAGVGWTFYPPLSGS.MYS.GR
Brachydistomum_sp__PakPr2_QIX04645 LPLFLGLRDLSLPRMNSLSVWLLGPSTLCLSTSMF..CGAGVGWTFYPPLSSL.GYS.GV
Brachylecithum_sp__PakAb2_QIX04633 LPLFLGLKDLSLPRMNSMSVWLLGPSTICLASSMF..GGAGVGWTFYPPLSSS.LYS.GW
Dicrocoelium_dendriticum_AHG06504 LPLFLGLSDLSLPRMNALSVWLLGPASFCLSTSMF..WGAGVGWTFYPPLSGS.GYS.GW
Dicrocoelium_chinensis_AHG06492 LPLFLGLSDLSLPRVNALSVWLLGPASFCLAASMF..GGAGVGWTFYPPLSGL.GYS.GW
Eurytrema_coelomaticum_ON297668 MPLFLGLDDLSLPRMNALSVWLLAPACICLVVSMF..GGAGVGWTFYPPLSSS.SYS.GW
Eurytrema_pancreaticum_AKC58423 MPLFLGLNDLSLPRMNALSVWLLVPSCICLVISMF..GGAGVGWTFYPPLSSS.GYS.GW
Schistosoma_spindale_YP_626439 LPFFLYLDDLLLPRLNSFSLWLMIPSLFYMELSLY..YGCGVGWTLYPPLSII.EGS.GL
Schistosoma_haematobium_YP_626526 LPFFLYIDDLLLPRLNSFSLWLMIPSFFYMELSLY..YGCGVGWTLYPPLSIS.ENS.GL
Schistosoma_mekongi_AAG12187 LPFFLCLDDLALPRLNSFSVWVMVPSIFYMELSLY..YGSGVGWTFYPPLSSQ.ATS.GV
Schistosoma_japonicum_AAG13143 LPFFLSMSDLALPRLNSLSVWMMVPSIFYMELSLY..YGSGVGWTFYPPLSSL.ATS.GV
Cotylurus_marcogliesei_AYH51378 LPLLLGMPDLSLPRLNALSAWLMLPSVVCFGISLS..MGSGVGWTFYPPLSSF.PYS.GV
Prosthogonimus_cuneatus_YP_009938512 LPLLLGKDDLDLPRLNGLSLWLLLPSSFCLGVSMF..LGAGVGWTFYPPLSSF.DYS.GR
Diplostomum_ardeae_YP_009867153 LPLLLGLPDLSLPRLNALSAWLMLPSAVCFIISLW..IGSGVGWTFYPPLSSF.PYT.GV
Paragonimus_westermani_NP_059465 LPLLLGIPDLSLPRLNALSAWLMLPSAVCLSLSML..GGAGVGWTFYPPLSGG.DYS.GW
Paramphistomum_cervi_YP_008963803 LPLLLGLPDLILPRLNALSAWLLLPSVVCLCLSLV..KGAGVGWTFYPPLSGG.EFSSGH
Ogmocotyle_sikae_YP_009139763 LPLLLGIPDLNLPRLNALSAWLILPASVCLALSMF..LGSGVGWTFYPPLSGG.DYSVGH
Fasciola_hepatica_COX1_15504 LPLLLGIPDLNLPRLNALSAWLLFPACVCLPFGLI..GGVGVGWTFYPPLSSL.DYS.SW
Fasciola_gigantica_YP_009026843 LPLLLGIPDLNLPRLNALSAWLLLPACVCLSFGLM..GGMGVGWTFYPPLSSL.DYS.GW
Fischoederius_elongatus_YP_009169430 LPLLLGLPDLILPRINALSAWLLLPSTVCLCLSLV..KGAGVGWTFYPPLAGG.EFSTGH
```

```
                                          150       160       170       180       190       200
Homo_sapiens_P00395                   SVDLTIFSLHLAGVSSILGAINFITTIINMKPP.AMTQYQTPLFVWSVLITAVLLLLSLP
Caenorhabditis_elegans_P24893         SVDLAIFSLHAAGLSSILGGINFMCTTKNLRSS.SISLEHMTLFVWTVFVTVFLLVLSLP
Ascaris_suum_P24881                   SVDLAIFSLHCAGVSSILGAINFMTTTKNLRSS.SISLEHMSLFVWTVFVTVFLLVLSLP
Lyperosomum_longicauda_QIX04657       GVDFLMFSLHLAGVSSLLSSLNFITTVLEVMDW..VCFLRLSIIVWGYLFTSVLLVLSLP
Brachydistomum_sp__PakPr2_QIX04645    GVDFLMFSLHLAGVSSIFSSINFVSSVAEALDR..ISFGRHSIIVWAYLFTAVLLIMSLP
Brachylecithum_sp__PakAb2_QIX04633    GVDFLMFSLHLAGVSSVFSSINFISSVGEVLDR..ISLGRHSIIVWAYLFTAVLLVMSLP
Dicrocoelium_dendriticum_AHG06504     GVDFLMFSLHLAGVSSILSSINFICSVFEVLDR..VTLGRHSIIVWAYLFTAILLVMSLP
Dicrocoelium_chinensis_AHG06492       GVDFLMFSLHLAGVSSILGSINFICSVFEALDR..VTFGRHSIVVWAYLFTAILLVMSLP
Eurytrema_coelomaticum_ON297668       GVNFLMFSLHLAGLSSIFGSLNFISTIIGASDR..VAVSRHSIIVWAYLFTSVLLIMSLP
Eurytrema_pancreaticum_AKC58423       GVNFLMFSLHLAGLSSIFGSLNFISTIIGASDR..VVISRHSIIVWAYLFTSVLLIMSLP
Schistosoma_spindale_YP_626439        GVDYLMFSLHLAGVSSLIGSINFISTIVSRIN......LSSSIIIWAYLFTSILLLLSLP
Schistosoma_haematobium_YP_626526     GVDYLMFSLHLAGVSSLVGSINFISTIISRVD......FKTSIIIWSYLFTSILLLLSLP
Schistosoma_mekongi_AAG12187          GVDYLMFSLHLAGISSLIGSVNFITTIMIRLN......SCSSVISWSYFFTSILLLISLP
Schistosoma_japonicum_AAG13143        GVDYLMFSLHLAGVSSLIGSINFITTIMLRLS......SCSSVISWSYLFTSVLLLISLP
Cotylurus_marcogliesei_AYH51378       GVDYLMFSLHLAGLSSVLGSLNFITTIFSSIFF..FINTRVSIIVWAYLFTSFLLLFSLP
Prosthogonimus_cuneatus_YP_009938512  GVDFLMFSLHLAGVSSLLGSLNFISTICGVMSYGKHVCFRLSIVVWSYLFTSILLVLSLP
Diplostomum_ardeae_YP_009867153       GVDYLMFSLHLAGLSSVFGALNFVTTIFSSIFY..FINTRVSIIVWAYLFTSILLLSSLP
Paragonimus_westermani_NP_059465      GVDFLMFSLHLAGLSSLLGSLNFICTIVSALCE..HTVGRSSIIVWAYLFTSVLLLLSLP
Paramphistomum_cervi_YP_008963803     GVDFLMFSLHLTGISSILSSLNFIATIYSAVNV..YTSSRQSVLVWAYLFTSILLVLSLP
Ogmocotyle_sikae_YP_009139763         GADFMMFSLHLAGVSSILSSLNFICTIYGGVED..WNASRQSIIVWAYLFTSVLLIISLP
Fasciola_hepatica_COX1_15504          GVDFLMFSLHLAGVSSLLGSINFICTILEVMLD..EGTGRHSILVWAYLFTSVLLLLSLP
Fasciola_gigantica_YP_009026843       GVDFLMFSLHLAGVSSLLGSINFICTILEVMLG..EGTGRHSILVWAYLFTSILLLLSLP
Fischoederius_elongatus_YP_009169430  GVDFLMFSLHLAGVSSILSSLNFIATIYSAVNI..YTSSRQSVLVWAYLFTSILLILSLP

                                          210       220       230       240       250       260
Homo_sapiens_P00395                   VLAAGITMLLTDRNLNTTFFDPAGGGDPILYQHLFWFFGHPEVYILILPGFGMISHIVTY
Caenorhabditis_elegans_P24893         VLAGAITMLLTDRNLNTSFFDPSTGGNPLIYQHLFWFFGHPEVYILILPAFGIVSQSTLY
Ascaris_suum_P24881                   VLAGAITMLLTDRNLNTSFFDPSTGGNPLIYQHLFWFFGHPEVYILILPAFGIISQSSLY
Lyperosomum_longicauda_QIX04657       VLAAAITMLLFDRNFGSSFFDPSGGGDPVLFQHLFWFFGHPEVYVLIIPGFAVVSHVCMS
Brachydistomum_sp__PakPr2_QIX04645    VLAAAITMLLFDRNFASAFFDPSGGGDPVLFQHLFWFFGHPEVYVLIIPGFGMVSHVCMC
Brachylecithum_sp__PakAb2_QIX04633    VLAAAITMLLFDRNFASAFFDPSGGGDPILFQHLFWFFGHPEVYVLIIPGFGMVSHVCMG
Dicrocoelium_dendriticum_AHG06504     VLAAAITMLLFDRHFASAFFDPAGGGDPVLFQHLFWFFGHPEVYVLIIPGFGMVSHVCMC
Dicrocoelium_chinensis_AHG06492       VLAAAITMLLFDRHFASAFFDPAGGGDPVLFQHLFWFFGHPEVYVLIIPGFGMVSHVCMC
Eurytrema_coelomaticum_ON297668       VLAAAITMLLFDRNFASSFFDPSGGGDPVLFQHLFWFFGHPEVYVLIIPGFGIVSHVCLS
Eurytrema_pancreaticum_AKC58423       VLAAAITMLLFDRNFASSFFDPSGGGDPVLFQHLFWFFGHPEVYVLIIPGFGIVSHICLS
Schistosoma_spindale_YP_626439        VLAAGITMLLFDRNFGTAFFEPTGGGDPLLFQHLFWFFGHPEVYVLILPGFGIVSHICMM
Schistosoma_haematobium_YP_626526     VLAAGITMLLFDRNFGTAFFEPMGGGDPLLFQHLFWFFGHPEVYVLILPGFGIVSHICMS
Schistosoma_mekongi_AAG12187          VLAAGITMLLFDRNFGTAFFEPAGGGDPVLFQHLFWFFGHPEVYVLILPGFGIVSHICMT
Schistosoma_japonicum_AAG13143        VLAAGITMLLFDRNFGTAFFEPAGGGDPVLFQHLFWFFGHPEVYVLILPGFGIVSHICMS
Cotylurus_marcogliesei_AYH51378       VLAAAITMLLFDRNFSSSFFDPTGGGDPILFQHMFWFFGHPEVYVLILPGFGMISHICLI
Prosthogonimus_cuneatus_YP_009938512  VLASGITMLLFDRNFGSSFFDPLGGGDPVLFQHLFWFFGHPEVYVLILPGFGIVSHVCSS
Diplostomum_ardeae_YP_009867153       VLAAAITMLLFDRNFGSSFFDPVGGGDPILFQHMFWFFGHPEVYVLILPGFGMISHICLV
Paragonimus_westermani_NP_059465      VLAAAITMLLFDRNFSSAFFDPLGGGDPVLFQHLFWFFGHPEVYVLILPGFGIVSHICMT
Paramphistomum_cervi_YP_008963803     VLAAGITMLLFDRNFGTSFFDPLGGGDPVLFQHLFWFFGHPEVYVLILPGFGVVSHICMS
Ogmocotyle_sikae_YP_009139763         VLAAGITMLLFDRNFESSFFDPLGGGDPVLFQHLFWFFGHPEVYVLILPAFGIVSHICLS
Fasciola_hepatica_COX1_15504          VLAAAITMLLFDRNFGSAFFDPMGGGDPVLFQHLFWFFGHPEVYVLILPGFGVISHICVT
Fasciola_gigantica_YP_009026843       VLAAAITMLLFDRNFSSAFFDPMGGGDPVLFQHLFWFFGHPEVYVLILPGFGVISHICMT
Fischoederius_elongatus_YP_009169430  VLAAGITMLLFDRNFGTSFFDPLGGGDPVLFQHLFWFFGHPEVYVLILPGFGAVSHICMC

                                          270       280       290       300       310       320
Homo_sapiens_P00395                   YSGKKEPFGYMGMVWAMMSIGFLGFIVWAHHMFTVGMDVDTRAYFTSATMIIAIPTGVKV
Caenorhabditis_elegans_P24893         LTGKKEVFGALGMVYAILSIGLIGCVVWAHHMYTVGMDLDSRAYFSAATMVIAVPTGVKV
Ascaris_suum_P24881                   LTGKKEVFGSLGMVYAILSIGLIGCVVWAHHMYTVGMDLDSRAYFTAATMVIAVPTGVKV
Lyperosomum_longicauda_QIX04657       LINNDSLFGYMGLVFAIVSIVCLGSVVWAHHMFVVGLDVKTAVFFSSVTMIIAVPTGVKV
Brachydistomum_sp__PakPr2_QIX04645    VTNNDSLFGYTGLVFALVAIVCLGSIVWAHHMFVVGLDFKTVIFFSSVTMIIGVPTGIKV
Brachylecithum_sp__PakAb2_QIX04633    LTNNDSLFGYLGLIFAMVAIVCLGSVVWAHHMFVVGLDVKTVIFFSSVTMIIGVPTGIKV
Dicrocoelium_dendriticum_AHG06504     VTNNDSLFGYMGLVFALVAIVCLGSIVWAHHMFMVGLDFKTVIFFSSVTMIIGVPTGIKV
Dicrocoelium_chinensis_AHG06492       VTNNDSLFGYMGLVFAMVAIVCLGSIVWAHHMFMVGLDFKTVIFFSSVTMIIGVPTGIKV
Eurytrema_coelomaticum_ON297668       FTNNDSLFGYLGLVFAIVAIVCLGSVVWVHHMFVVGLDVKTAVFFSSVTMIIGVPTGIKI
Eurytrema_pancreaticum_AKC58423       FTNNDSLFGYLGLVFAIVAIVCLGSVVWVHHMFVVGLDVKTAVFFSSVTMIIGVPTGIKI
Schistosoma_spindale_YP_626439        ISNNDSSFGYYGLICAMASIVCLGSVVWAHHMFMVGLDSLTAVFFSSVTMIIGIPTGIKV
Schistosoma_haematobium_YP_626526     ISNNDSSFGYYGLICAMASIVCLGSVVWAHHMFMVGLDYLTAIFFSSVTMIIGIPTGIKV
Schistosoma_mekongi_AAG12187          LSNNNSLFGYYGLVCAMGSIVCLGSVVWAHHMFMVGMDVKTAIFFSSVTMIIGIPTGIKV
Schistosoma_japonicum_AAG13143        LSNNNSSFGYYGLVCAMGSIVCLGSVVWAHHMFMVGMDVKTSVFFSSVTMIIGIPTGIKV
Cotylurus_marcogliesei_AYH51378       FTNNDSVFSYMGLVGAMFSIVVLGFIVWVHHMFMVGLEFRSLVFFSSTTMVIGIPTGIKV
Prosthogonimus_cuneatus_YP_009938512  LSTSKGVFGYLGMVFAMGSIVCLGSVVWGHHMFMVGFDLQTTVFFSSVTMIIGIPTGIKV
Diplostomum_ardeae_YP_009867153       FTNNDSVFSYTGLVGAMFSIVILGCIVWVHHMFMVGLEFRSLVFFSSTTMVIGIPTGIKV
Paragonimus_westermani_NP_059465      LTNNDSLFGYYGLVFAMGAIVCLGSVVWAHHMFMVGLDVKTAVFFSSVTGVIGIPTGIKV
Paramphistomum_cervi_YP_008963803     LSNQDSLFGYYGLVFAMASIVCLGSVVWAHHMFMVGLDVKTSVFFSSVTMVIGIPTGIKV
Ogmocotyle_sikae_YP_009139763         LSNSDSLFGYFGLVFAMASILCLGSVVWAHHMFMVGLDVKTAVFFSSVTMVIGIPTGIKI
Fasciola_hepatica_COX1_15504          LTNNDSLFGYYGLILAMAAIVCLGSVVWAHHMFMVGLDVHTAVFFSSVTMVIGIPTGIKV
Fasciola_gigantica_YP_009026843       LTNNDSLFGYYGLILAMAAIVCLGSVVWAHHMFMVGLDVHTAVFFSSVTMVIGIPTGIKV
Fischoederius_elongatus_YP_009169430  LSNQDSLFGYYGIVFAMASIVCLGSVVWAHHMFMVGLDVKTSVFFSSVSMVIGIPTGIKV
```

```
                                                       330       340       350       360       370
Homo_sapiens_P00395                      FSWLATLHG.SNM.KWSAAVLWALGFIFLFTVGGLTGIVLANSSLDIVLHDTYYVVAHFH
Caenorhabditis_elegans_P24893            FSWLATLFG.MKM.VFNPLLLWVLGFIFLFTLGGLTGVVLSNSSLDIILHDTYYVVSHFH
Ascaris_suum_P24881                      FSWLATLFG.MKM.VFQPLLLWVMGFIFLFTIGGLTGVMLSNSSLDIILHDTYYVVSHFH
Lyperosomum_longicauda_QIX04657          FSWLYMLSH.GSVRLWDPVIWWIGGFIFLFTIGGITGIILSASVLDGTLHDTWFVVAHFH
Brachydistomum_sp__PakPr2_QIX04645       FSWLYMLGC.GSVRLWDPVIWWIGGFIGLFTIGGVTGIMLSASVLDTLLHDTWFVVAHFH
Brachylecithum_sp__PakAb2_QIX04633       FSWLYMLGC.SGIRLWDPVIWWVGGFIFLFTVGGITGVILSASVLDSALHDTWFVVAHFH
Dicrocoelium_dendriticum_AHG06504        FSWLYMLSC.SGVRLWDPVVWWVGGFIFLFTVGGITGVVLSASILDGVLHDTWFVVAHFH
Dicrocoelium_chinensis_AHG06492          FSWLYMLGC.GGVRLWDPVVWWVGGFIFLFTVGGITGVILSASVLDGVLHDTWFVVAHFH
Eurytrema_coelomaticum_ON297668          FSWLYMLGN.SNSRLWDPVVWWIAGFIFLFTVGGITGIVLSASVLDGALHDSWFVVAHFH
Eurytrema_pancreaticum_AKC58423          FSWLYMLGN.SNSRLWDPVVWWIAGFIFLFTVGGITGIVLSASVLDGALHDSWFVVAHFH
Schistosoma_spindale_YP_626439           FSWLFMLNS.CGTRVSDPVLWWLVGFIFLFVIGGVTGVALSSSALDVLFHDTWFVVAHFH
Schistosoma_haematobium_YP_626526        FSWLYMLNS.CGSRVWDPIVWWLVGFIFLFTIGGVTGIALSASSLDILFHDTWFVVAHFH
Schistosoma_mekongi_AAG12187             FSWLYMLSG.CGLRVIDPVVWWIVGFIFLFTVGGVTGIVLSASSLDSLFHDTWFVVAHFH
Schistosoma_japonicum_AAG13143           FSWLYMLGS.SGLRAADPILWWIVGFIFLFTVGGVTGIVLSASALDSLFHDTWFVIAHFH
Cotylurus_marcogliesei_AYH51378          FSWIYMLRG.CWFRIMDPIFWWILGFIFLFTIGGVTGIMLSASVLDSIFHDTWFVIAHFH
Prosthogonimus_cuneatus_YP_009938512     FSWLYMLSG.CNTSLNNPLVWWVIGFIFLFTIGGVTGIALSASVLDSLLHDSWFVVAHFH
Diplostomum_ardeae_YP_009867153          FSWLYMLRG.AWYRMNEPIFWWILGFIFLFTIGGVTGIMLSASVLDSIFHDTWFVIAHFH
Paragonimus_westermani_NP_059465         FSWLFMLGG.TRLRFWDPVLWWILGFIFLFTIGGVTGIILSSSILDSLLHDTWFVVAHFH
Paramphistomum_cervi_YP_008963803        FSWLYMLSG.TSVRIWDPVVWWIMGFIVLFTIGGVTGIVLSSCVLDSMVHDTWFVVAHFH
Ogmocotyle_sikae_YP_009139763            FSWLYMLSG.SSIRSGDPVLWWIIGFIILFTIGGVTGIVLSASVLDTIVHDTWFVVAHFH
Fasciola_hepatica_COX1_15504             FSWLIMLGGGSSVRIWDPVVWWIIGFIVLFTIGGVTGIMLSASLLDTLLHDTWFVVAHFH
Fasciola_gigantica_YP_009026843          FSWLIMLGGGSSVRIWDPVVWWIVGFVVLFTIGGVTGIMLSASLLDTLLHDTWFVVAHFH
Fischoederius_elongatus_YP_009169430     FSWLYMLSG.VGVRAWDPVVWWIMGFIILFTMGGVTGIVLSSCVLDSMVHDTWFVVAHFH

                                         380       390       400       410       420       430
Homo_sapiens_P00395                      YVLSMGAVFAIMGGFIHWFPLFSGYTLDQTYAKIHFTIMFIGVNLTFFPQHFLGLSGMPR
Caenorhabditis_elegans_P24893            YVLSLGAVFGIFTGVTLWWSFITGYVLDKLMMSAVFILLFIGVNLTFFPLHFAGLHGFPR
Ascaris_suum_P24881                      YVLSLGAVFGIFTGVTLWWSFITGFVYDKMMMSSVFVLMFVGVNLTFFPLHFAGIHGYPR
Lyperosomum_longicauda_QIX04657          YVLSLGSYSTVIISVLWWWPSMIGTSLSKYLVEGHWLVSMLGFNLCFFPMHYLGVCGLPR
Brachydistomum_sp__PakPr2_QIX04645       YVLSLGSFSTVMVSLLWWWPGVIGVSLNKYLLQGHWISSMFGFNLCFFPMHYLGICGLPR
Brachylecithum_sp__PakAb2_QIX04633       YVLSLGSYSTVIVSLLWWWPGVIGVSLNKYLLESHWFSSMFGFNLCFFPMHYLGTWGLPR
Dicrocoelium_dendriticum_AHG06504        YVLSLGSFSTVIIALLWWWPGVIGVSLNKYLLEGHWFSSMLGFNLCFFPMHYLGVWCVPR
Dicrocoelium_chinensis_AHG06492          YVLSLGSFSTVIIALLWWWPSVVGVSLNKYLLEGHWFSSMLGFNLCFFPMHYLGVWCVPR
Eurytrema_coelomaticum_ON297668          YVLSLGSYSTVVISILWWWPSVVGVSLNKYLLEGHWIASMFGFNLCFFPMHYLGTSGLPR
Eurytrema_pancreaticum_AKC58423          YVLSLGSYSTVIISILWWWPGVVGASLNKYLLQSHWFASMFGFNLCFFPMHYLGTSGLPR
Schistosoma_spindale_YP_626439           YVLSLGSYSSVVIMLVWWWPFVIGLSLNKYLLQGHWLLSMVGFNLCFFPMHYLGIHGLPR
Schistosoma_haematobium_YP_626526        YVLSLGSYSSVVIMLLWWWPFIIGYSINKYLLQGHWLLSMVGFNLCFFPMHYLGIHGLPR
Schistosoma_mekongi_AAG12187             YVLSLGSYSSLIIMILWWWPFIIGCSLNKYLLQGHWLLSMVGFNLCFFPMHYLGMHGLPR
Schistosoma_japonicum_AAG13143           YVLSLGSYSSVIIMFVWWWPFIIGCSLNKYLLQGHWLLSMVGFNLCFFPMHYLGVHGLPR
Cotylurus_marcogliesei_AYH51378          YVLSLGSYITVVIALIWWWPVISGFSLDNVLLYAHWFVSMVGFNLCFFPMHYSGMCGLPR
Prosthogonimus_cuneatus_YP_009938512     YVLSLGSYSSVVIYLLWGWPMISGNSVNKSLVQGHWISSMIGFNFCFFPMHFMGLFGLPR
Diplostomum_ardeae_YP_009867153          YVLSLGSYSTVVIALVWWWPIISGYVLDKTLLFGHWLVSMIGFNLCFFPMHYTGMYGLPR
Paragonimus_westermani_NP_059465         YVLSLGSYSTVVISLLWWWPVVTGFSLNKYLLQGHWVSSMLGFNLCFFPMHYLGAYGLPR
Paramphistomum_cervi_YP_008963803        YVLSLGSYSAVVISLLWWWPLVVGVSLNKYLLQGHWLCSMVGFNLCFFPMHYFGLCGLPR
Ogmocotyle_sikae_YP_009139763            YVLSLGSYSSVVISIIWWWPVITGLSLNKYLLQGHWLCSMVGFNLCFFPMHYLGMCGLPR
Fasciola_hepatica_COX1_15504             YVLSLGSYSSVVISFIWWWPVVTGYSLNLFMLQGHWVVSMIGFNMCFFPMHYLGMAGLPR
Fasciola_gigantica_YP_009026843          YVLSLGSYSSVVISFIWWWPIVTGYSLNLFMLQGHWFVSMVGFNMCFFPMHYLGMCGLPR
Fischoederius_elongatus_YP_009169430     YVLSLGSYSALVISIIWWWPLIVGYSLNKYMLQGHWLCSMLGVNLCFFPMHYFGLCGLPR

                                         440       450       460       470       480       490
Homo_sapiens_P00395                      RYSDYPDAYTTWNILSSVGSFISLTAVMLMIFMIWEAFASKRKVLMVEEPSMNLEWLYGC
Caenorhabditis_elegans_P24893            KYLDYPDVYSVWNIIASYGSIISTAGLFLFIYVLLESFFSYRLVISDYYSNSSPEYCMSN
Ascaris_suum_P24881                      KYLDYPDVYSVWNIMASYGSMISVFALFLFIYVLLESFVGHRIFLFDYYVNSGPEYSLSG
Lyperosomum_longicauda_QIX04657          RVSSFDSMFHWLNSFSSLGAVLSVISAFFFFFLVWESMFVCNRVIGCWGSCGVPLNLFTV
Brachydistomum_sp__PakPr2_QIX04645       RVSNFDSGFHWLHSFSSLGGIISVVSSFFFFFLVWESMVICNRVIGCWGSATVPFNVFTV
Brachylecithum_sp__PakAb2_QIX04633       RVSSFDSGFFWLNGFSSLGGILSVVSAFFFFFLVWESMAICNRVIGCWGSACVPFNLFTV
Dicrocoelium_dendriticum_AHG06504        RASSFDSSFFWLNGLSSLGGVLSLVSAFFFFFLVWESIVIGNRVIGCWGSASVPFNVFTI
Dicrocoelium_chinensis_AHG06492          RASSFDSSFFWLNGFSSLGGVLSLVSAFFFFFLVWESIVIGNRVIGCWGSASVPFNVFTV
Eurytrema_coelomaticum_ON297668          RVSSFDSGFFWLNSFSSLGALLSVISAFFFFFLVWESMFICNRVIGCWGSCSSPINLFTV
Eurytrema_pancreaticum_AKC58423          RVSSFDSGFFWLNSFSSLGALLSVVSAFFFFFWFGN.LCLFVIVLLVVG...........
Schistosoma_spindale_YP_626439           RVSCYDSEYYWINVLSSMGSVMSVLSAFILVFILWESLSVGNRVIGLWGSNSSVTNVMTL
Schistosoma_haematobium_YP_626526        RVSSYDFEYYWINVLSSIGGVLSVTSAFVLFFILWESLSVCNRIIGLWGSGSCVTNVMVV
Schistosoma_mekongi_AAG12187             RVSCYDPEFYVVNVFSTIGGILSVTSSMVFMFIIWESLSVFNRILGLWGPGSCICNVVTI
Schistosoma_japonicum_AAG13143           RVSCYDPEFYLVNVFSSIGGVLSVTSSMVFMFLLWESLSVYNCVLGLWGSCGCVCNVLTC
Cotylurus_marcogliesei_AYH51378          RVCVYDPLFYKLNVISNFGGLLSVISGFFLIYIVWDSMYYGNSVIASYGTSAVVLYLVTL
Prosthogonimus_cuneatus_YP_009938512     RVCSFDPCFIWLNSFSSWGALISVCSAFFLLFLFWESLVVGHRVLGVWGDSLLSVHLKTV
Diplostomum_ardeae_YP_009867153          RVCVYDPAFYKLNIISNFGGLLSVMSAFFLFYIVWHSYEFGSSVIGVYGSSAMVLYLVTL
Paragonimus_westermani_NP_059465         RVCNYEPAFQWLNNISSVGALVSVVSAFFLVFILWESLVVGNRVVGIWGTNALVVNALVT
Paramphistomum_cervi_YP_008963803        RVCVYNPDFFWLESLASFGAFLSVISAFFLVFILWESLVVSSVVVAAWGSSNVSLNVVVL
Ogmocotyle_sikae_YP_009139763            RVCVYDLSFSWINSIMSLGGLISVVSGFFLVFLLWESLTVQNKVFFVWGSNNLIFNVVSV
Fasciola_hepatica_COX1_15504             RVCVYDPDFYWLSVVSSLGALVSAGSAFLFVFILWESFVVGNCVVSSWGSSSLVLNVITL
Fasciola_gigantica_YP_009026843          RVCVYDPDFYWLSVVSSVGAFISAGSAFLFLFVLWESFVVGNRVVSSWGNSSLVLNVITL
Fischoederius_elongatus_YP_009169430     RVCVYNPDFYWLESLSSFGAFLATISAFFLVLILWESLVVGNVVVAAWGSSNVSLHVVAL
```

```
                                      500               510
                                        .                 .
Homo_sapiens_P00395                   PPPYH.....TFEEPV.YM.........................................
Caenorhabditis_elegans_P24893         YVFGHSYQSEIYFSTT.SL.........................................
Ascaris_suum_P24881                   YVFGHSYQSEIFYSSI.VF.........................................
Lyperosomum_longicauda_QIX04657       PLAHHI...GWFNYPTRWS.........................................
Brachydistomum_sp__PakPr2_QIX04645    PVSHHA...DWFDRPVRWL.........................................
Brachylecithum_sp__PakAb2_QIX04633    PLAHHV...HWFDHPTRW..........................................
Dicrocoelium_dendriticum_AHG06504     SIAHHV...HWFERPVRWL.........................................
Dicrocoelium_chinensis_AHG06492       SIAHHV...HWFERPVRWL.........................................
Eurytrema_coelomaticum_ON297668       PVAYHV...GWFCRPCRWF.........................................
Eurytrema_pancreaticum_AKC58423       ...............VLLG.........................................
Schistosoma_spindale_YP_626439        PVPFHI...SYICGGKVWFG........................................
Schistosoma_haematobium_YP_626526     PVPVHI...TYICGGKVWFN........................................
Schistosoma_mekongi_AAG12187          PVPSHI...GYMVNGKYWTW........................................
Schistosoma_japonicum_AAG13143        PTSYHI...DYIADGKNW..........................................
Cotylurus_marcogliesei_AYH51378       PLPHHG...TYISSPTYYVSREVALNFLLKRD...........ND............SKH
Prosthogonimus_cuneatus_YP_009938512  PFEHHE...EFSKDPTYFIGWS..............KVEVDWYSLCLGYLKSVWDYVVGV
Diplostomum_ardeae_YP_009867153       PLPHHC...TYMSLPRYYISDLISLSFFPNEAVEKNKKKITWRQL............LLS
Paragonimus_westermani_NP_059465      PVPHHS...IYISGPSRWL.........................................
Paramphistomum_cervi_YP_008963803     PVPQHV...AYMSGSSRW..........................................
Ogmocotyle_sikae_YP_009139763         PVPQHT...SYISGPSRWIFS.......................................
Fasciola_hepatica_COX1_15504          PGPQHN...SYMNGVGRWV.........................................
Fasciola_gigantica_YP_009026843       PGPQHN...SYMNGAGRWV.........................................
Fischoederius_elongatus_YP_009169430  PVPQHV...TYMSGSNRW..........................................


Homo_sapiens_P00395                   ....KS
Caenorhabditis_elegans_P24893         ....KN
Ascaris_suum_P24881                   ....KF
Lyperosomum_longicauda_QIX04657       ....NF
Brachydistomum_sp__PakPr2_QIX04645    ....PW
Brachylecithum_sp__PakAb2_QIX04633    ....SS
Dicrocoelium_dendriticum_AHG06504     ....AW
Dicrocoelium_chinensis_AHG06492       ....LW
Eurytrema_coelomaticum_ON297668       ....GF
Eurytrema_pancreaticum_AKC58423       ....LL
Schistosoma_spindale_YP_626439        ...YCI
Schistosoma_haematobium_YP_626526     ....YK
Schistosoma_mekongi_AAG12187          ...GNK
Schistosoma_japonicum_AAG13143        ...SKY
Cotylurus_marcogliesei_AYH51378       NASSKF
Prosthogonimus_cuneatus_YP_009938512  YKSFKY
Diplostomum_ardeae_YP_009867153       LFSKSE
Paragonimus_westermani_NP_059465      ....SF
Paramphistomum_cervi_YP_008963803     .....F
Ogmocotyle_sikae_YP_009139763         ..LGNV
Fasciola_hepatica_COX1_15504          .....F
Fasciola_gigantica_YP_009026843       .....F
Fischoederius_elongatus_YP_009169430  .....F
```
