# Supplementary figures and images for: Characterization of the Complete Mitochondrial Genome and Phylogenetic Analyses of Eurytrema coelomaticum (Trematoda: Dicrocoeliidae)
Source: Genes (Basel). 2023 Dec 11;14(12):2199. doi: 10.3390/genes14122199 (PMC10743053; doi:10.3390/genes14122199)

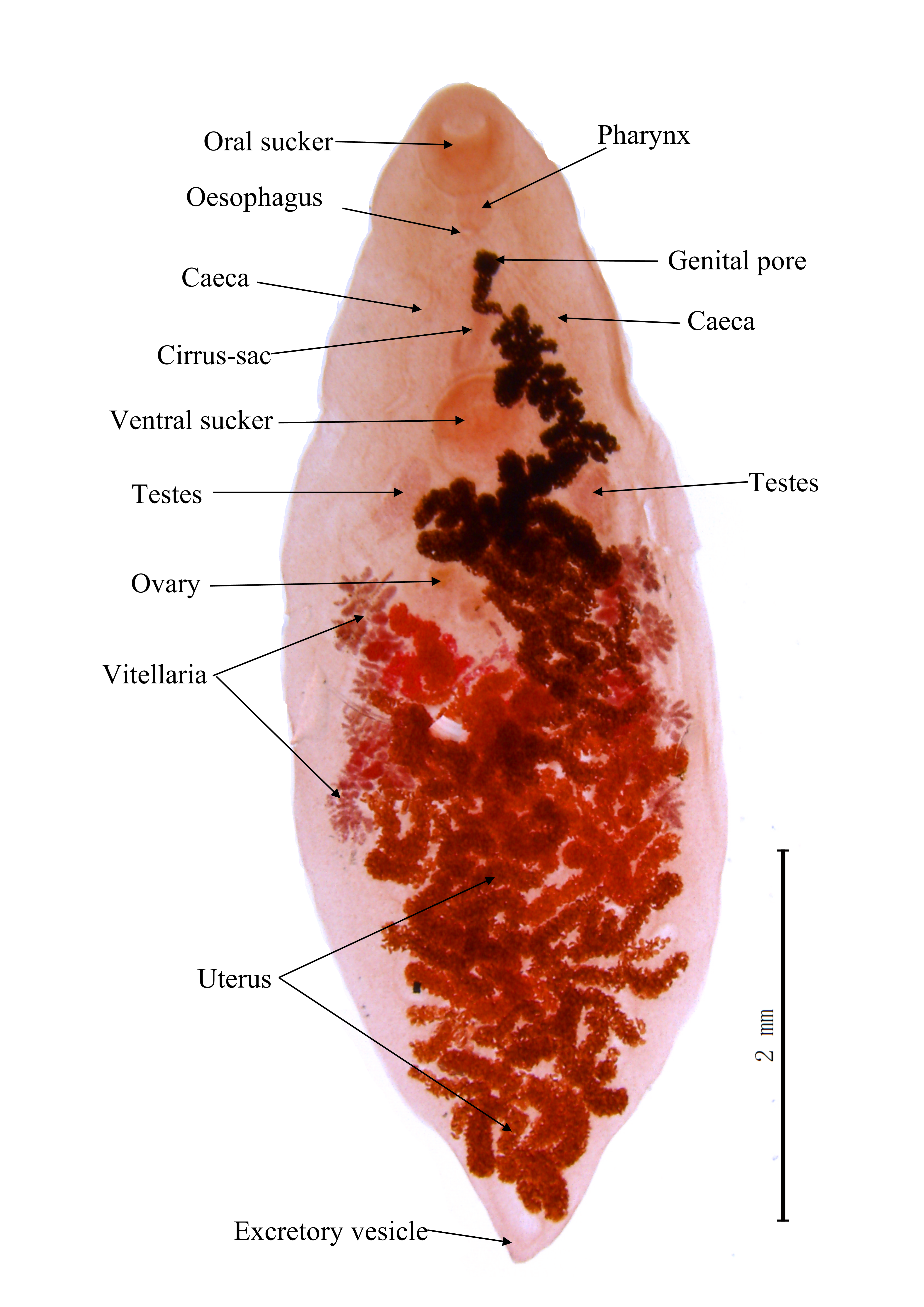

Supplement: Supplementary file 1 [file genes-14-02199-s001.zip › Figure S1.tif]

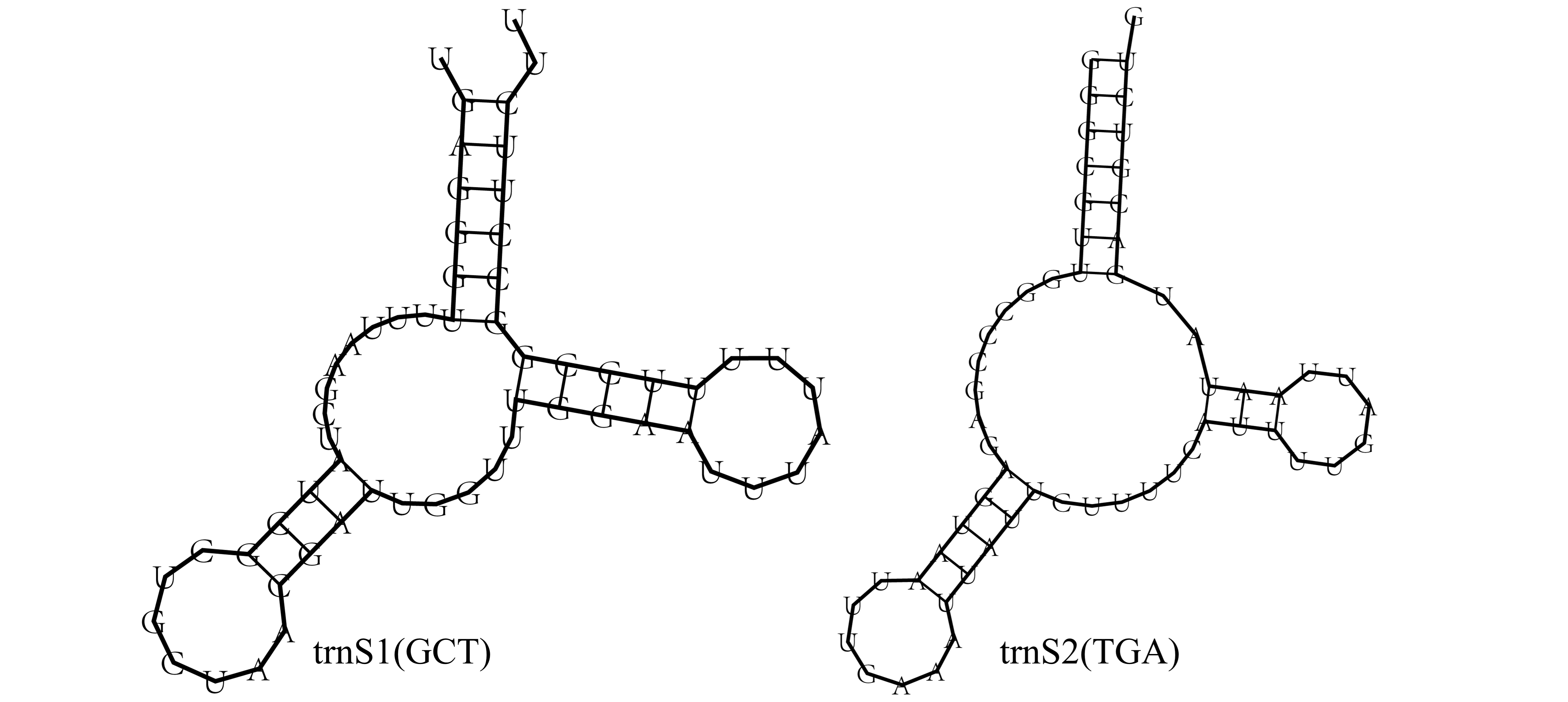

Supplement: Supplementary file 1 [file genes-14-02199-s001.zip › Figure S2.tif]
